# Fully integrated Monte Carlo simulation for evaluating radiation induced DNA damage and subsequent repair using Geant4-DNA

**DOI:** 10.1038/s41598-020-75982-x

**Published:** 2020-11-27

**Authors:** Dousatsu Sakata, Oleg Belov, Marie-Claude Bordage, Dimitris Emfietzoglou, Susanna Guatelli, Taku Inaniwa, Vladimir Ivanchenko, Mathieu Karamitros, Ioanna Kyriakou, Nathanael Lampe, Ivan Petrovic, Aleksandra Ristic-Fira, Wook-Geun Shin, Sebastien Incerti

**Affiliations:** 1 Department of Accelerator and Medical Physics, National Institute of Radiological Sciences, QST, Chiba, Japan; 2 Joint Institute for Nuclear Research, Dubna, Russia; 3 Dubna State University, Dubna, Russia; 4 INSERM, UMR 1037, CRCT, Université Paul Sabatier, Toulouse, France; 5 UMR 1037, CRCT, Université Toulouse III-Paul Sabatier, Toulouse, France; 6 Medical Physics Laboratory, Medical School, University of Ioannina, 45110 Ioannina, Greece; 7 Centre For Medical Radiation Physics, University of Wollongong, Wollongong, Australia; 8 Geant4 Associates International Ltd, Hebden Bridge, UK; 9 Tomsk State University, Tomsk, Russia; 10 Unaffiliated, Bordeaux, France; 11 Unaffiliated, Melbourne, Australia; 12 Vinca Institute of Nuclear Science, University of Belgrade, Belgrade, Serbia; 13 Univ. Bordeaux, CNRS, CENBG, UMR 5797, Gradignan, 33170 France

**Keywords:** Computational science, Cell biology, Medical research, Chemistry, Physics

## Abstract

Ionising radiation induced DNA damage and subsequent biological responses to it depend on the radiation’s track-structure and its energy loss distribution pattern. To investigate the underlying biological mechanisms involved in such complex system, there is need of predicting biological response by integrated Monte Carlo (MC) simulations across physics, chemistry and biology. Hence, in this work, we have developed an application using the open source Geant4-DNA toolkit to propose a realistic “fully integrated” MC simulation to calculate both early DNA damage and subsequent biological responses with time. We had previously developed an application allowing simulations of radiation induced early DNA damage on a naked cell nucleus model. In the new version presented in this work, we have developed three additional important features: (1) modeling of a realistic cell geometry, (2) inclusion of a biological repair model, (3) refinement of DNA damage parameters for direct damage and indirect damage scoring. The simulation results are validated with experimental data in terms of Single Strand Break (SSB) yields for plasmid and Double Strand Break (DSB) yields for plasmid/human cell. In addition, the yields of indirect DSBs are compatible with the experimental scavengeable damage fraction. The simulation application also demonstrates agreement with experimental data of $\gamma$-H2AX yields for gamma ray irradiation. Using this application, it is now possible to predict biological response along time through track-structure MC simulations.

## Introduction

Modeling of ionising radiation induced DNA damage and subsequent biological responses such as DNA repair processes is still a challenging issue due to the complexity of the problem. The response of a living cell when exposed to ionising radiation can be measured by means of biological assays quantifying reproductive cell death and other biological markers such as the cell survival curve^1,2^, DNA fragment yields using electrophoresis^3,4^ and accumulation of DNA-repair protein through *foci* detection^5–7^. According to experimental findings, biological endpoints ascribed to the mutation triggered by DNA damage strongly depend on incident particle type and energy. These dependencies are, typically, discussed as a function of Linear Energy Transfer (LET) of the radiation field^8^. From a more microscopic point of view, it is known that the complexity of DNA damage affects its lethalness. The damage complexity is related to the microscopic energy deposition pattern in the region surrounding DNA molecules as discussed for many years in microdosimetric/nanodosimetric studies^9–12^. Ionising radiation induced DNA damage is created by direct interaction of DNA molecules with particles and indirect interaction of DNA with molecular species created during water radiolysis (typically reactive oxygen species such as hydroxyl radicals)^13^. However, a comprehensive understanding of how ionising radiation leads to biological endpoints, across multi-scale radiobiological phenomena, is yet to be reached. In conventional approaches, to model biological response bridging a gap between DNA damage and biological endpoints, DNA damage yield and its lethalness are usually estimated by empirical models^14,15^. Such models are not directly connected to track structures themselves. Ideally, in order to fully understand radiation induced DNA damage, a mechanistic model associating track structure of radiation with biological endpoints is required. Such a platform should be equipped with abilities to evaluate not only the yield of DNA damage but also the detailed description of the damage, for instance, the fraction of direct/indirect damage induced by physical/chemical interactions respectively as well as the complexity of DNA damage^16^.

Monte Carlo (MC) simulation codes such as KURBUC^10,17,18^, PARTRAC^19–22^ and Geant4-DNA^23–26^, which is a low energy extension of the MC toolkit Geant4^27–29^, are useful to evaluate the number of DNA damage events and their complexity induced by an ionising radiation field. These MC simulations are able to calculate physical, physico-chemical and chemical interactions^30,31^. Recently, using Geant4-DNA, an integrated simulation application has been developed to evaluate proton induced DNA damage in a human cell nucleus model composed of fractally distributed chromatin fibre^32^ (called hereafter Geant4-DNA_2019) and including an efficient modelling of radiolysis, proposing an alternative to the previous Geant4-DNA works by Meylan et al.^33^ (called hereafter Geant4-DNA_SM) and by Rosales et al.^34^. The simulations using Geant4-DNA_2019 have been validated against experimental DNA damage^35–40^ by means of electrophoresis, e.g. agarose gel electrophoresis (AGE)^3^ and pulsed-field gel electrophoresis (PFGE)^4^, and against previous MC simulations (KURBUC, PARTRAC)^10,20,33^. With DNA damage models similar to the ones of PARTRAC, Geant4-DNA_2019 demonstrated almost equivalent simulation results to PARTRAC^32^ which is considered to be one of the state-of-the-art MC codes for radiation induced DNA damage.

However, until now, track-structure MC simulations are not able to predict biological response and endpoints from their simulated early DNA damage. As the last piece of developing a multi-scale platform for radiobiology, one current challenge in MC simulations is to model and predict biological responses in MC simulations^41,42^. Hence, in this work, we have upgraded the Geant4-DNA_2019 simulation application to propose a realistic “fully integrated” MC simulation combining physics, chemistry and biology, to predict biological responses in MC simulations.

## Materials and methods

We have applied three major upgrades to the Geant4-DNA_2019 simulation application to propose a realistic “fully integrated” MC simulation including the new features (see “New features for fully integrated MC simulation” *a-c* section). In the following sections, after introduction of the new features, we briefly summarise the simulation configuration (“Simulation configuration” section), damage scoring/classification (“Damage scoring and classification” section), biological repair model (“Biological repair model” section), as well as verification/validation (“Verification and validation” section). All developments and simulations are based on Geant4 version 10.4.patch2.

## New features for fully integrated MC simulation

### a. Building a realistic cell geometry by wrapping the cell nucleus in cytoplasm

In the previous MC application^32^, a naked cell nucleus was simulated for evaluating radiation induced DNA damage, however in reality, the cell nucleus is immersed in cytoplasm and surrounded by the cell membrane. Hence in this work, we have built a more realistic cell model (see “Simulation configuration” section), placing an ellipsoidal water absorber around the cell nucleus in order to model the cytoplasm.

### b. Introducing biological repair model

The previous application could only calculate early DNA damage within a few nano seconds after irradiation. In this work we extended the functionality of the simulation by introducing a semi-empirical model to predict the *foci* accumulation yield along time, up to 25 h after irradiation (see “Biological repair model” section). The model was developed by Belov et al.^15^ and requires the yield of Double Strand Break (DSB) and the fraction of complex DNA damage (see “Damage scoring and classification” section) as input.

### c. Refinement of model parameters for direct damage and indirect damage scoring

The third upgrade is a re-adjustment of the model parameters to calculate direct and indirect damage. For the re-adjustment, in addition to considering DSB yield, we introduce damage patterns of plasmids, the indirect damage fraction and DNA repair (see “Verification and validation” section) as criteria to determine more realistic model parameters.

As a criterion of the re-adjustment, we performed simulations without the histone scavenging function in order to validate the simulation against the simple DNA fibre of plasmids. Because of experimental difficulties in measuring the yield of Single Strand Breaks (SSBs) for mammalian cells, the previous MC simulations were validated for DSB yields only (and/or fragment distributions separated by a pair of DSBs). Instead, for plasmids, it is possible to measure both the number of SSB and number of DSB for validation, even though the base-pair (bp) density of plasmids is smaller than the bp density of mammalian cell (c.f. $\rho _{plasmid} \sim 9.4\times 10^{-6}~\mathrm {bp/nm}^{3}$, $\rho _{cell} \sim 1.5 \times 10^{-2}~\mathrm {bp/nm}^{3}$^43,44^), and the structure of circular plasmid DNA fibres is twisted forming a supercoil. Hence, in this work, we performed simulations with and without the histone scavenging function in order to obtain a validation against both mammalian chromosome and simple DNA fibre of plasmid. Also, for the validation of indirect damage fraction, we simulate the fraction of indirect DSBs and compare to it the experimental scavengeable damage fraction estimated as the maximum scavengeable cell death fraction at infinite hydroxyl scavenger concentration.

In accordance with our previous works^45,46^, to perform more realistic simulations, the chemical stage is modelled for an extended period of time up to 5 ns, limiting the simulation time to reasonable value (see “Simulation configuration” section), which is two times longer than the chemical stage duration considered in Geant4-DNA_2019.

### Simulation configuration

**Figure 1:**
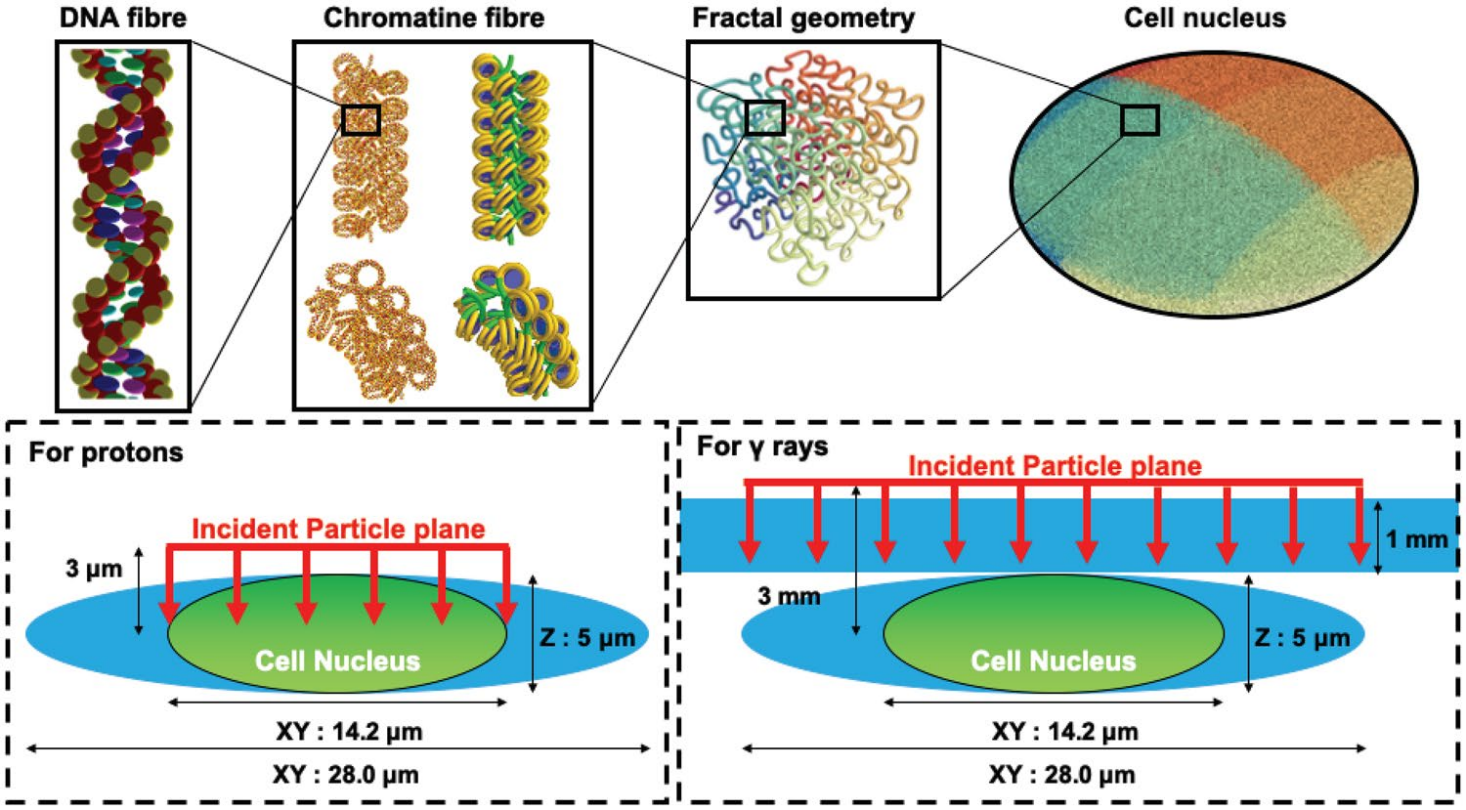
Geometrical model of cell nucleus and its sub-structure. Double helix DNA (left top) is wrapped by histone forming chromatin fiber (2nd left top, left: double helix DNA/right: DNA fibre and histone). Chromatin fibres are assembled as fractal (3rd left top) and confined in ellipsoidal cell nucleus (4th left top). Materials surrounding cell nucleus and beam geometry are shown in bottom panels, for both protons and gamma rays.

The cell nucleus geometry, used in this work, has been developed in Geant4-DNA_2019^32^. The simulation geometry is presented in Fig. 1. A unit base pair (bp) of DNA is composed of six molecules consisting of two pairs of spherical phosphate molecules (${\hbox {H}}_3 {\hbox {PO}}_4$) and spherical deoxyribose molecules with two ellipsoidal nucleotide bases (guanine, adenine, cytosine and thymine) as a backbone. The combination of bases (adenine-thymine/cytosine-guanine) of each nucleobase pair is chosen randomly. As shown in left top panel of Fig. 1, these DNA units compose DNA fibre forming a twisted structure as known as double-helix structure with a $34^\circ$ rotation angle per base pair^47–49^. In the cell nucleus model, the DNA fibre is folded compactly by spherical histones and confined in a cube 75 nm on each side (shown in 2nd left top panel of Fig. 1), forming the fractal base chromatin fibre geometry (shown in 2nd right top panel of Fig. 1). Applying spherical or ellipsoidal mask to the piled up fractal chromatin geometry, it is possible to define any cell nucleus geometrical model (shown in right top panel of Fig. 1). The number of base pairs accommodated in typical human fibroblast cell is around 6 Gbp within a nucleus volume of $\sim 500~\upmu {\text {m}}^{3}$ ($\rho _{fibroblast} \sim 0.012~\text {bp/nm}^{3}$)^50,51^. The typical volume of fibroblast cells is around $2000~\upmu {\text {m}}^{3}$^51^. Hence, in this work, a $14.2~\upmu \mathrm {m} \times 14.2~\upmu \mathrm {m} \times 5.0~\upmu \mathrm {m}$ long ellipsoidal cell nucleus ($\sim ~528 ~\upmu {\text {m}}^{3}$) is surrounded by an ellipsoid of water ($28.0~\upmu \mathrm {m} \times 28.0~\upmu \mathrm {m} \times 5.0~\upmu \mathrm {m}$, $\sim 2052~\upmu {\text {m}}^{3}$) modelling the cytoplasm. The total number of base pairs is around 6.4 Gbp and the corresponding bp density in the cell nucleus is approximately $0.012~{\hbox {bp/nm}}^{3}$. Experimentally, a substrate made of glass or polymer is placed downstream of beam, on which cells are platted. To simulate particle transport in the substrate, we need to know the specific details of the substrate, such as size, composition of material, density and mean excitation energy (so called I-value). In general, it’s difficult to know such details if they are not provided. However, in the literature, the particle energies (excluding gamma rays) and their unrestricted LET at the cell entrance have been already estimated^35–40^. Likewise, in the corresponding literature about scavengeable damage fractions, the particle energies (excluding X-rays) and their dose averaged LET have been estimated^52,53^. This is the reason why, in this work, we did not place any substrate in the simulations for proton irradiation to fairly compare the simulated results against the experimental results on DSB yields and scavengeable damage fraction (as shown in left bottom panel of Fig. 1). For gamma rays, neither particle energy spectra at the cell entrance or material detail of the substrate are provided. In the *foci* experiment^54^, the cells are platted in a Falcon  T25 cell culture flask, however, it is difficult to access the details of substrate. Hence, as an alternative approach, we place a 1 mm thick water absorber just before of the cell geometry for gamma ray irradiation (shown in right bottom panel of Fig. 1).

The simulations are performed by evaluating DNA damage in a cell nucleus irradiated by mono-energetic protons. The initial energies of protons are chosen to be 0.3, 0.4, 0.7, 1.0, 1.67, 2.34, 4.0, 7.0 and 50 MeV. They correspond to a wide range of unrestricted linear energy transfer (${\hbox {LET}}_\infty$) between 1.2 and 54.41 $\hbox {keV/}\upmu \hbox {m}$. The lowest energy of incident protons is higher than the limit of Geant4-DNA_2019 (0.1 MeV), due to the unreliability of the application in the very high LET domain. The simulations are repeated for gamma rays emitted by a $^{60} \hbox {Co}$ source (energy equal to 1.17 MeV and 1.33 MeV with the same frequency) and a $^{137} \hbox {Cs}$ source (energy equal to 661.7 keV, 32.1 keV and 36.5 keV with the frequency of about 0.92, 0.06 and 0.01, respectively). The radiation source is homogeneously distributed on a $14.2 \,\upmu \hbox {m}$ diameter circle plane which corresponds to the 2D-profile of the ellipsoidal cell nucleus for DSB yields and scavengeable fraction (shown as red line in left bottom panels of Fig. 1) , and a $28 \, \upmu \hbox {m}$ diameter circle plane which corresponds to the 2D-profile of the ellipsoidal cell for *foci* yield to keep the uniformity of radiation field at the cell nucleus level (shown as red line in right bottom panels of Fig. 1).

DNA geometries are defined as liquid water absorber with scaled density of the corresponding biological material similarly as in our previous work^32^. G4EmDNAPhysics_option4^55–59^ has been used to model the particle interactions in the cell for protons, photons, and low energy electrons below 10 keV, since the underlying model provides an improved implementation of the dielectric response of liquid water which is important at low energies. Above 10 keV, for electron interactions, G4EmDNAPhysics_option2 is utilized since the high energy limit of G4EmDNAPhysics_option4 for electrons is 10 keV. These physics lists adopt a track structure approach which is important when describing particle interactions at nano scale level^60^. Geant4-DNA provides an interface for chemistry simulations^61,62^. The production and reaction schemes of radiolysis for chemical species are utilized in the default configuration of the Geant4-DNA chemistry with a newly developed independent-reaction time (IRT) method^63,64^ which allows for fast-diffusion control of the radiolysis species instead of the default step-by-step method. We model histones as perfect scavengers for all radiolysis species. This means that, in the simulations, any free radiolysis species that enters a histone region (modelled as a 2.5 nm radius sphere) will be stopped and terminated. Additionally, we kill all radiolysis species created more than 9.0 nm away from any DNA molecule centres (${\hbox {d}}_{kill}^{chem}$), in order to reduce computing time since they will likely be scavenged, i.e., the probability of interactions of DNA molecules and the radiolysis species is small. This range cut is equivalent to the maximum diffusion distance of hydroxyl radical (which could cause indirect damage) at 5.0 ns (${\hbox {T}}_{chem}$), since all simulations are stopped at 5.0 ns after the diffusion started. These limits of chemical diffusion are summarized in Table. 1.

**Table 1:** Damage parameters and chemistry limits of this work and previous MC simulations.

	KURBUC^18^	PARTRAC^20^	Geant-DNA_SM^33^	Geant4-DNA_2019^32^	This Work
$R_{dir}$ (Å)	$1.7\hbox {-}3.25^{\heartsuit }$	${\hbox {2VDWR}}^{\diamondsuit }$	VDWR $^{\star }$	4.5	3.5
$E_{min}^{break}$ (eV)	17.5	5	17.5	5	5
$E_{max}^{break}$ (eV)	17.5	37.5	17.5	37.5	37.5
$P_{OH}^{break}$	0.13	$0.7^{\,\spadesuit }$	0.4	0.4	0.405
$T_{chem}$ (ns)	1	$10^{\,\clubsuit }$	2.5	2.5	5
$d_{kill}^{chem}$ (nm)	4	$12.5^{\,*}$	N/A	4.5	9

$R_{dir}$: Accumulation radius of energy deposition from nucleotide centre. $E_{min}^{break}$: Minimum energy of direct strand break probability model. $E_{max}^{break}$: Maximum energy of direct strand break probability model. $P_{OH}^{break}$: Probability of indirect strand break. $T_{chem}$: Time limit of chemical diffusion. $d_{kill}^{chem}$: Production range limit of chemical radiolysis species from nucleotide centre. (VDWR) Summing up of atomic volume with each atomic van der Waals Radius (1.2, 1.7, 1.5, 1.4, 1.9 Å for H, C, N, O, P respectively). ($\heartsuit$) Arch structure with $1.7\times 3.25~\AA$ section. ($\diamondsuit$) To Adjust for cross-section of molecules and take into account hydration shell, VDWR multiplied by 2. ($\spadesuit$)Only damage with deoxyribose is considered as indirect damage. ($\clubsuit$) Additional 2.5 ns hydroxyl radical scavenging is considered. ($*$) Distance from centre of chromatine fibre. ($\star$) Additionally 24 water molecules considered as hydration shell.

Overall, total computing time for 100 incident protons of 10 MeV is about 15 h including geometry building ($\sim$5 h) using Intel Xeon CPU E5-2630 v2 (2.60 GHz). It’s almost double compared to Geant4-DNA_2019 ($\sim$7 h in total), and the time difference is getting longer in the high LET domain. On the other hand, Geant4-DNA_SM requires approximately 10 days^33^ for similar simulation conditions using step-by-step chemistry without production limit distance of chemical species (${\hbox {d}}_{kill}^{chem}$).

### Damage scoring and classification

The damage scoring model and the classification method of DNA damage were originally proposed by Nikjoo et al.^18^. The direct DNA damage model was upgraded in PARTRAC^20^ using a more realistic damage probability by means of a linearly increasing function of energy deposition, substituting the threshold probability model used in KURBUC. In order to calculate direct/indirect DNA damage in Geant4-DNA_2019, the same models of PARTRAC were used^20^.

Estimating the model parameters related to probabilities of direct damage and indirect damage is one of the most uncertain part of such simulations. The model parameters of this work are summarized in Table 1 and compared to other MC codes. As listed in the table, the parameters vary covering a wide range. Usually, these parameters are adjusted within a reasonable range. For instance, PARTRAC assumes the total probability of direct damage adjusting the radius of nucleotide ($R_{dir}$ 1.3–1.4 times larger than the nucleotide size)^20^. Another assumption of PARTRAC is that it considers the semi-bounded 10 water molecules surrounding nucleotide^20^, so called hydration shell^65–67^, which allows direct damage by charge transfer on these water molecules. Overall, $R_{dir}$ used in PARTRAC simulations is two times more than the nucleotide size. Compared to Geant4-DNA_2019, we additionally used plasmid-SSB, plasmid-DSB, scavengeable fraction and *foci* accumulation as criteria for the adjustment of the parameters.

To calculate the probability of direct strand breaks in this work, first of all, all energy deposition was assigned to the closest strand molecule when the position is within 3.5 Å from the centre of a strand molecule in the case of either sugar or phosphate ($R_{dir}$). This distance is larger than the radius of nucleotide molecules ($R_{phosphate} \sim 2.28~\AA$ and $R_{deoxyribose} \sim 2.63~\AA$) to take into account charge transfer effect in hydration shell, and smaller than the size used in PARTRAC simulations. After each simulation of the tracking of an incident particle and associated secondary radiation field, the total accumulated energy deposit in scoring region is used to determine the probability that a strand break occurred along the DNA fibres. Based on a linearly increasing probability distribution, there is a 0% probability a break occurred when less than 5 eV ($E_{min}^{break}$) was deposited, but a 100% probability a break occurred when over 37.5 eV ($E_{max}^{break}$) was deposited in the sugar phosphate moiety, as done in PARTRAC simulations.

The number of indirect damage depends on the value chosen for the likelihood of a chemical reaction between a hydroxyl radical and the sugar phosphate backbone leads to a single strand break (SSB). As the result of adjustment, to increase indirect damage, the probability of indirect damage ($P^{break}_{OH}$) is set to 0.405 which is almost the same (within $\sim$ 1%) with the value used in Geant4-DNA_2019, and smaller than PARTRAC simulations.

**Figure 2:**
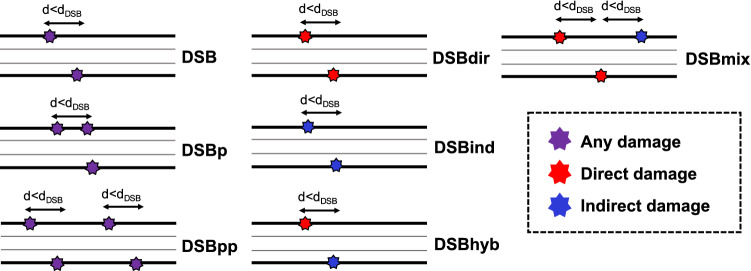
The scheme of classification for complexity of DSB (left) and for source of DSB (centre/right)^31,47^.

In the present work, all strand breaks (SBs) are classified either as direct SB or indirect SB. Usually, early DNA damage is quantified as number of DSBs which is considered as being two SBs on opposite strands within short distance between each other. The complexity of DSB is also scored when quantifying DNA damage. The complexity model for DSB entails a parameter $d_{DSB}$ which is the maximum separation length between two damage sites on opposite sides of a DNA strand to consider that a DSB has occurred (typically $d_{DSB} = 10 \, \hbox {bp}$). The complexity types are shown left panel of Fig. 2. A DSBp (DSB plus) requires a DSB and at least one additional break within $d_{DSB}$, while a DSBpp (DSB plus-plus) requires at least two DSBs along the chromatin fibre segment. Since the lengths of segment are different (KURBUC: 216 bp, This work: straight: 7330 bp; turn and turn-twisted: 5023 bp), we made a constraint with interval of bp length. It will identify a separate damage as occurring when there is a sequence of unbroken DNA strictly greater than 100 bp between two damage events. For DSB complexity, the most complex break type is always selected for the damage cluster. Nikjoo et al.^18^ also proposed the classification scheme of DSB breaks by direct/indirect damage source as shown in the middle and the right panel of Fig. 2, since the contribution of chemically scavengeable DNA damage is also of particular interest. When classifying breaks by source, we pay attention to not only damage type, but also to the damage sources. DSBs composed by two indirect damage are classified as DSBind, and those only by direct damage are classified as DSBdir. DSBhyb requires that the DSB doesn’t occur in the absence of indirect damage when a segment contains both indirect and direct DSBs. Otherwise, a break caused by indirect and direct sources is classified as DSBmix. Similarly, when a segment contains a DSB composed by both direct and indirect damage, classified as DSBhyb in conjunction with a direct damage for classification.

### Biological repair model

In this work, we introduced a semi-empirical theoretical model for predicting biological repair effect presented by Belov et al.^15^. The repair model computes yields of accumulated proteins for four principal repair pathways to repair DSBs, namely non-homologous end-joining (NHEJ), homologous recombination (HR), single-strand annealing (SSA), and alternative end-joining mechanism (Alt-NHEJ).

In these considerations, the kinetics of DSB induction can be calculated using the number of complex DSB (${\hbox {N}}_{cDSB}$) and of non-complex DSB (${\hbox {N}}_{ncDSB}$) by^15^

1 $$\begin{aligned} \frac{dN_{0}}{dt} = \alpha (L)\frac{dD}{dt}N_{cDSB}-V_{NHEJ}-V_{HR}-V_{SSA}-V_{microSSA}-V_{AltNHEJ}, \end{aligned}$$

where $N_{0}=N_{ncDSB}+N_{cDSB}$; $V_{NHEJ}$, $V_{HR}$, $V_{SSA}$, $V_{microSSA}$, and $V_{AltNHEJ}$ are the terms characterizing elimination of DSBs by the NHEJ, HR, SSA, micro-SSA, and Alt-NHEJ repair pathways respectively. *D* is the dose of ionising radiation (Gy) and $\alpha (L)$ is the slope coefficient of linear dose dependence which describes DSB induction per unit of dose (${\hbox {Gy}}^{-1}$ per cell) and depends on LET. To simulate the processing of DNA lesions by repair enzymes the mass-action kinetics approach is used. The kinetics of DSB induction and remaining DSB is simulated using differential equation (1) describing a balance between increase (term $\alpha (L)\frac{dD}{dt}N_{cDSB}$) and decrease of DSB level due to the action of all the repertoire of the repair pathways. This model includes dose rate (*dD*/*dt*) effect during irradiation, however in this work, it is assumed that the dose is delivered with very high dose rate, then fixed $dD/dt=D$ at $t=0$ and $dD/dt=0$ at $t \ne 0$ as in the study by Belov et al.^15^. For each repair path, the DSB elimination terms are expressed as differential equation with the rate constants of the correspond protein attachment and disattachment involved in the repair processes. A dynamic change of intracellular concentrations of main intermediate complexes can be generally expressed by the following differential equation:

2 $$\begin{aligned} \frac{dX}{dt}=V_{+}(X_{i},N_{0}) - V_{-}(X_{i},N_{0}) \end{aligned}$$

where $X_{i}~(i=1,...,n)$ is the intracellular level of the $i$-th attachment/disattachment processes of each pathway, *t* is time, *n* is the total number of processes, the functions $V_{+}$ and $V_{-}$ describe the complex attachment and disattachment, respectively. $V_{+}$ is the summation of intracellular protein concentrations multiplied by each protein reaction rate constant at the $i$-th process. Similarly $V_{-}$ is the summation of intracellular protein degradation. The dimensionless form of the system of differential equations refers to calculation of each pathway as well as to its rate constants of target protein. More details are described in the work by Belov et al.^15^ In total, the repair model has 53 rate constants along the repair paths. Most of the rate constants are evaluated by fitting to the experimental data on kinetics of different stages of DSB repair^15^. Solving the multi differential equations, the yields of target protein along time are calculated. In this work, five *foci* accumulation yields can be calculated for Ku, DNA-PKcs, RPA, Rad51 and $\gamma$-H2AX, although, we mainly discuss in terms of *foci* signal from $\gamma$-H2AX using specific immunocytochemistry antibodies raised against $\gamma$-H2AX. Using $\gamma$-H2AX *foci* as a biomarker of radiation exposure, the repair time-curve is estimated by means of the quantification of *foci* per nucleus at corresponding times after irradiation^5^. When DNA damage arises forming DSB, it is usually followed by the phosphorylation of the variant H2AX of the H2A protein family, which is a component of the histone octamer in nucleosomes. It is phosphorylated by kinases such as phosphatidyl inosito-3 kinase-related kinase (PIKK) family of proteins which include DSB repair enzymes, ataxia telangiectasia mutated (ATM), DNA-PKcs and ATM and RAD3-related (ATR)^68^, and the phosphorylated H2AX is called $\gamma$-H2AX. According to past studies, major contribution of radiation-induced $\gamma$-H2AX *foci* formation is DNA-PKcs and activated ATM^69^. Hence, *foci* induction by Michaelis–Menten kinetics is calculated for the time-curve by summing up all active forms of DNA-PKcs and ATM which remain in defective cells as either DNA-PKcs or LigIV after irradiation.

In this work, it is assumed that the number of complex DSB can be calculated by $N_{cDSB}=N_{DSBp}+2\times N_{DSBpp}$, where $N_{DSBp}$ is the number of DSBp and $N_{DSBpp}$ is number of DSBpp, though DSBpp could contain more than two DSBs (the number of complex DSBs composed by three DSBs and more DSBs is neglected by definition). In order to obtain these curves, we used the NHEJ, HR, SSA, and Alt-NHEJ models with zero initial conditions for all intermediate complexes and corresponding values of cDSB.

### Verification and validation

The simulation platform is verified against past MC studies and validated against experimental measurements for both protons and gamma rays. Before classifying DNA damage clusters into either SSB or DSB, the total number of strand breaks and their contributions to direct/indirect damage are compared to the past MC simulations for verification. After the DNA damage clusters are classified, both SSB and DSB yields are validated against experimental measurements and benchmarked against the past MC simulations^18,20,32,33^, followed by validation of scavengeable DNA damage fraction and time-curve of *foci* yield.

Since the cell geometry is modeled imitating a normal human fibroblast cell, the experimental results for normal human fibroblast cells are chosen for validation of the DSB yields and the *foci* yields. In addition, as a reference, we have compared simulated DSB yields in simple DNA fibre against experimental DSB yields of plasmid. For scavengeable damage fraction, normal Chinese hamster fibroblast cell (V79) is selected for the validation, because it is one of the most popular cell lines in radiobiology, and a systematic experimental study of scavengeable damage fraction for human fibroblast cell is not available. Compared to human fibroblast cell, V79 is smaller (average diameter of nucleus $\sim \, 8 \, \upmu \hbox {m}$ and cell $\sim \, 10 \, \upmu \hbox {m}$^70^) and number of base pairs is smaller ($\sim$ 3.9 Gbp^40^), but the base pair density is higher ($\rho _{V79} \sim \, 0.015~{\hbox {bp/nm}}^{3}$).

Experimentally, there are mainly two ways to evaluate DSB yields. The first method is based on the separation of DNA fragments through AGE or PFGE. The method can be performed both with plasmid and cell. To compare simulation results to these experimental data both for plasmid and mammalian cell, we have tried to simulate DSB yields with/without histone scavenging function. The simulation without histone scavenging can be regarded as simulation for simple DNA fibre such as plasmid, despite the bp density is different. In addition, unlike in cells, it’s possible to quantify not only DSB yield but also SSB yield using plasmids easily. The SSB/DSB ratio provides more insight of the details of radiation induced DNA damage. As an experimental constraint of AGE and PFGE in cells is the difficulty to count DSBs in small fragments (typically shorter than 5–25 kbp), since the measurement is accompanied by the loss of small fragments when cells are embedded in gel plugs^35^. This is why, we simulate not only the total number of DSBs but also that of “distant” DSBs, those which are separated by at least 10 kbp gaps between two DSBs. The DSB yields measured by electrophoresis are shown as a function of unrestricted linear energy transfer (${\hbox {LET}}_\infty$), which is recommended by the ICRU 90^71^ excluding the case of gamma rays from $^{60} \hbox {Co}$. Radiation induced DNA damage is strongly related to the beam quality which is commonly quantified using (the unrestricted) LET. To validate DSB yields with high LET protons, ideally, we need to compare the simulated results against low energy proton irradiation experiments. However, experimentally, it is difficult to perform cellular irradiation experiments with such low energy protons. Hence in this study, as reference, we show the simulated DSB yields compared against the experimental results with helium at high LET. We note that, in the clinically relevant energy domain, it is well known that DSB yield is depending on the LET, despite that the LET dependence cannot entirely explain the observed biological effects^8^. In the case of gamma rays from $^{60}\hbox {Co}$, we assume LET = 0.5 keV/μm which is the upper-limit of LET for the $^{60}\hbox {Co}$ photons^39^. Likewise, the scavengeable damage fractions are also shown as a function of LET, since the experimental data is not for protons but for carbons and X-rays. In the literature of experiments measuring scavengeable damage fractions, incident energy and dose averaged LETs have been described, hence in this study, we converted the incident particle energy to ${\hbox {LET}}_\infty$ excluding the case of X-rays. For X-rays, we set the dose-averaged LET at $9.4~\hbox {keV}/\upmu \hbox {m}$ as listed in the literature^53^.

The second method is based on detection of *foci* representing the accumulation of proteins related to DSB repair process^5–7^. *Foci* measurement has become a standard to evaluate radiation induced DNA damage, however, the relation between *foci* yield and number of DSBs is still unclear^72–74^. Hence in this work, only experimental data by AGE and PFGE are compared to the simulated number of DSBs as benchmark, because of the difficulty of evaluating absolute values of DSB yields by *foci* measurements. Whilst *foci* measurements cannot be used to determine the number of DSBs, they can be used to evaluate DNA repair processes with respect to time. The calculated time-curve of protein accumulation which is newly implemented into the simulation application is compared with experimental $\gamma$-H2AX yield irradiated with gamma rays from a $^{137}\hbox {Cs}$ source.

The scavengeable damage fraction can be measured from the maximum degree of protection (*DP*) at infinite dimethylsulfoxide (DMSO) concentration. DMSO has been used as scavenger of radiolysis free radical species, in particular hydroxyl radical. DMSO is permeated among the Chinese hamster cell V79 before irradiation with different concentration up to 1.0 mol. The *DP* is calculated as follows:

3 $$\begin{aligned} DP = \frac{\ln SF_{0}-\ln SF_{x}}{\ln SF_{0}} , \end{aligned}$$

where $SF_{0}$ and $SF_{x}$ are the survival fraction at 0 and *x* mol of DMSO, respectively. A regression line can be plotted over the reciprocal *DP* via fitting to the experimental reciprocal *DP*s with several concentrations of DMSO by Eq. (4) to estimate intercept of the fitting function.

4 $$\begin{aligned} \frac{1}{DP} = k \cdot \frac{1}{x} + y_{\infty }, \end{aligned}$$

where *k* is the slope of the equation and $y_{\infty }$ is the intercept of the function giving the inverse of the maximum *DP* at infinite DMSO concentration ($1/x=0$). To compare the scavengeable damage fraction obtained by the maximum *DP*, we calculate the fraction of scavengeable DSBs which could not be classified as DSB without indirect damage by:

5 $$\begin{aligned} { \frac{N_{DSBind}+N_{DSBhyb}}{N_{DSBdir}+N_{DSBmix}+N_{DSBind}+N_{DSBhyb}},} \end{aligned}$$

where $N_{DSBdir}, N_{DSBind},N_{DSBmix}$ and $N_{DSBhyb}$ are the numbers of DSBdir, DSBind, DSBmix and DSBhyb, respectively. The calculations are presented as a function of unrestricted linear energy transfer (${\hbox {LET}}_\infty$). By using Nikjoo’s classification, we neglect the contribution of *DSBmix* towards the scavengeable damage fraction, since it cannot be clearly classified as scavengeable DSB or not.

## Results

**Figure 3:**
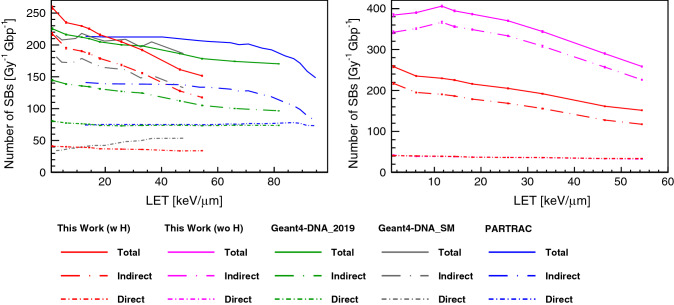
Number of strand breaks (SBs) per Gy and per Gbp induced by protons in a cell nucleus as a function of unrestricted LET. (Left: Comparison against the previous simulations. Right: Comparison with and without histone scavenging functionality). The solid lines show the total SB yield; the long dot-dashed lines show the indirect SB yield; the short lines show the direct SB yield. For this work, two types of histone scavenging conditions have been applied, simulation results with perfect scavenging (w H) are shown as red lines, and without scavenging functionality (wo H) are shown as magenta lines. The direct damage yields of this work with histone and without histone are overlapped with each other.

The total number of strand breaks (SBs) is plotted in Fig. 3 as a function of unrestricted LET, as well as the number of direct and indirect damage events. The yield of both direct and indirect damage is close to the yield of Geant4-DNA_SM in the low LET domain. The yield of indirect damage is gradually decreasing with LET for Geant4-DNA_SM. With the increase of LET, the total number of radiolysis species is increasing. Unlike indirect damage, there is no significant LET dependence in the case of direct damage.

When neglecting histone scavenging, the yield of indirect damage is strongly enhanced across the simulated LET range. For instance, the yield without histone scavenging functionality for radiolysis species at $10~\hbox {keV}/\upmu \hbox {m}$ is decreasing from around 350 down to $200~{\hbox {Gy}}^{-1} {\hbox {Gbp}}^{-1}$, since more hydroxyl radicals can survive if the radiolysis species are not scavenged by histone.

**Figure 4:**
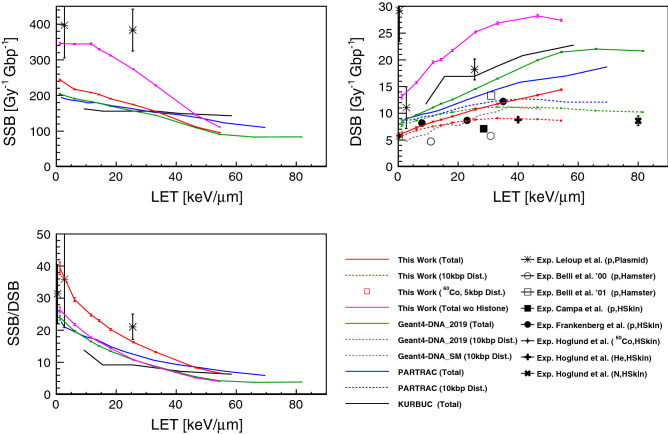
Left top: SSB yield per Gy and per Gbp as a function of unrestricted LET. Right top: DSB yield per Gy and per Gbp as a function of unrestricted LET. Left bottom: SSB/DSB ratio as a function of unrestricted LET. (HSkin: Human skin fibroblast cell). The simulated results are compared to the experimental data^35–40^.

Figure 4 shows the simulated SSB, DSB yields and their ratio as a function of unrestricted LET, including previous simulations^18,20,32^ and experimental results^35–39^. Similarly to the results deriving from the PARTRAC simulations, the simulated SSB yield is found to decrease with LET, although higher than PARTRAC at low LET (below $30 \, \hbox {keV}/\upmu \hbox {m}$) and smaller at high LET (above $40 \, \hbox {keV}/\upmu \hbox {m}$). If the histone scavenging effect is turned off, the SSB yield is getting larger, in particular at low LET, and the order of magnitude of SSB yield is reaching the yield level of the experimental SSB yield of plasmid.

As explained in “Verification and validation” section, to take into account experimental biases on the DSB yield, we calculate both the total DSB yield (solid red line) and the 10 kbp distant DSB yield (dotted red line) for proton irradiation. Also the 5 kbp distant DSB yield of gamma rays from $^{60} \hbox {Co}$ is presented as open red square where the limit of bp length is estimated from the experiment^39^. The total DSB yields are continually increasing with LET, while the difference between total DSBs and distant DSBs is also increasing because of the loss of small fragments in experimental procedure. The distant DSB yields for protons are approaching experimental data obtained by electrophoresis with a 13.3% difference on average. At the same time, the distant DSB yield for gamma rays is in agreement with the corresponding experimental data within 0.6%.

In terms of SSB/DSB ratio, the ratio decreases as a function of LET. This means that for higher LET values (above $40 \, \hbox {keV}/\upmu \hbox {m}$), radiation induced DNA damage becomes more significant because the DSB yield becomes bigger than the SSB yield.

**Figure 5:**
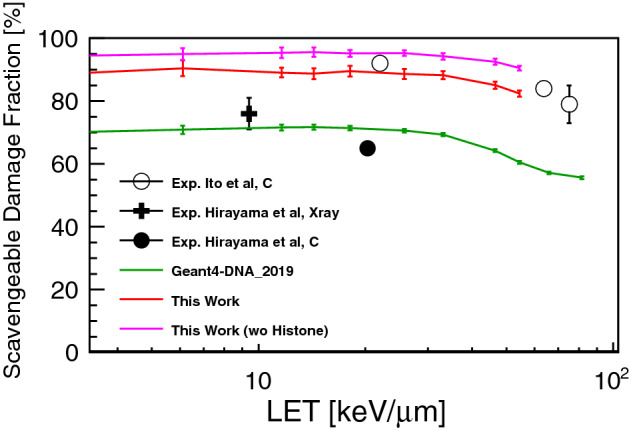
The scavengeable damage fraction by means of the ratio of scavengeable DSBs compared to the total number of DSBs. The simulated fraction is compared to the experimental scavengeable degree of V79 cell estimated at the infinite DMSO concentration^52,53^.

The proton induced scavengeable damage fraction is shown in Fig. 5. At low LET, about 90% of scavengeable damage fraction has been simulated, and with the increase of LET, the simulations indicate that the scavengeable damage fraction is decreasing. As in the simulations, the scavenging effect of histone reduces the predictable fraction by around 5% if the histone is regarded as a perfect scavenger. The simulated indirect DSBs are good agreement with the experimental scavengeable damage fraction^52^. Using previous damage parameters set used in Geant4-DNA_2019, it is possible to reproduce the other data set of the experimental scavengeable damage fraction^53^.

**Figure 6:**
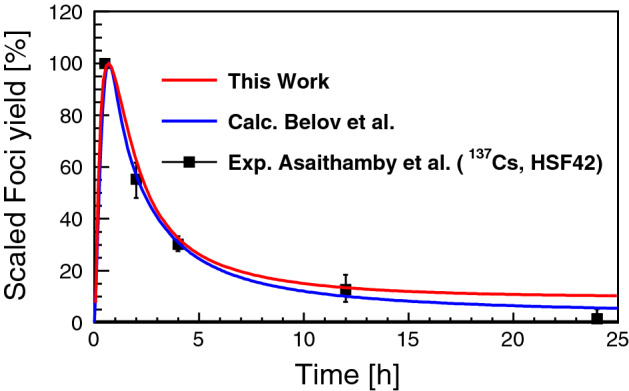
$\gamma$-H2AX yield as a function of time from irradiation by gamma rays from $^{137} \hbox {Cs}$ at the dose of 1 Gy. The predicted foci yield is calculated with simulated number of DSBs and complex DSBs as inputs. The experimental data is for normal human skin fibroblasts HSF42^54^.

Figure 6 shows the calculation results of scaled $\gamma$-H2AX yield as a function of time after irradiation up to 25 h with the simulated number of DSBs and the irreparable fraction as input parameters compared to the experimental data for normal human skin fibroblasts HSF42 exposed to gamma-rays from $^{137} \hbox {Cs}$ at the dose of 1 Gy^54^. The calculated $\gamma$-H2AX yield with optimized rate constants leads to a very good agreement with the experimental results (within 1.6% difference on average). The decreasing speed of $\gamma$-H2AX yield reasonably matches with the experimental result at 10 h.

## Discussion

The indirect SB yields of this work become lower than Geant4-DNA_SM with increasing LET, as shown in Fig. 3. With the increase of LET, the total number of radiolysis species is increasing, however, the yield of unstable species decreases quickly with time because of a higher reaction probability due to higher density around particle tracks. This is the reason why indirect SB yields become lower with increasing LET. However, in terms of direct SBs, the difference between the results of this work and Geant4-DNA_SM becomes larger with the increase of LET. As we discussed in the previous work^32^ the difference is mainly due to the probability model of direct damage and the size of the scoring volume^32^. It is more evident when comparing the direct yield calculated with this work and the yield deriving from PARTRAC simulations^20^, since the probability model is the same and the scoring volume of PARTRAC is larger than the volume adopted here (see Table 1).

The simulated yields of distant DSBs for protons and gamma rays from a $^{60} \hbox {Co}$ source are in good agreement with the experimental results (within 13.3% for protons on average and 0.7% for gamma rays) as shown in Fig. 4. In a previous study^47^, the systematic uncertainty from the physics particle transport model was estimated to be around 20% on electron induced DSB yields for simple DNA fibre. Similarly, using a cell nucleus model, physics model and chemistry model can cause uncertainties up to 34% and 16% in the simulated DSB yields respectively  as presented by Zhu et al.^75^. Also, direct damage model, chemistry time duration, and hydroxyl damage probability can cause differences of up to 28%, 51%, and 71%, respectively^75^. Therefore, the deviation from experimental data and the other simulations can be wrapped by the systematic uncertainties of the particle transport and it is not surprising that the DSB yields of this work are almost 20% lower than the yields of PARTRAC simulation. Compared to Geant4-DNA_2019, two improvements have been applied, firstly for geometry and secondly for simulation parameters. The difference between this work and Geant4-DNA_2019 mainly comes from the parameter refinement, the geometrical improvement having not much influence, (see Supplementary Figure S1 online which shows DSB yields of this work and Geant4-DNA_2019 with/without cytoplasm geometry.) Turning off the histone scavenging effect makes the SSB/DSB yield decrease. This suggest that histone scavenging has a role of protecting against radiation induced DSBs. PARTRAC simulates smaller SSB/DSB ratios compared to this work when considering histone scavenging. Hence, PARTRAC should simulate much smaller ratio if the histone scavenging effect is turned off. Compared to previous simulations^32^, this work is characterised by a better agreement to the experimental results in plasmid. It ought to be noted that we need to do further investigations of radiation induced DNA damage on plasmids with more realistic geometrical models ^76,77^ to have more fair comparisons (at least, comparison of simulations with more realistic plasmid bp density, since bp density affects number of indirect damage^45^).

When the scavengeable damage fraction is compared to the experimental data by Ito et al.^52^, the simulated fraction shows a good agreement in the LET range 20–54.41 keV/μm. However, since systematic differences can be found between the experiments by Ito et al.^52^ and by Hirayama et al.^53^, it’s difficult to get agreement with both experimental results at low and moderate LET (below a few tens $\hbox {keV}/\upmu \hbox {m}$). With the increase of base pair density, fraction of indirect damage is increasing^45^. Since the base pair density of V79 cell ($\rho _{V79}\sim \, 0.015 \, {\hbox {bp/nm}}^{3}$) is higher than the density of simulated cell nucleus model ($\rho _{fibroblast}\sim \, 0.012 \, {\hbox {bp/nm}}^{3}$), the experimental scavengeable damage fractions can be a few percent higher than the simulated scavengeable damage fractions. We note that further studies are required for simulations of scavengeable damage fraction with heavier ions for validation in the high LET domain. Also, a similar study for normal human fibroblast cells would be useful for a more comprehensive validation of this platform.

Across this work, the calculation results of scaled $\gamma$-H2AX yield have been validated for gamma-rays from $^{137} \hbox {Cs}$ at the dose of 1 Gy^54^. Both this work and calculation by Belov et al. are close to the experimental data below 5 h. After 5 h, this work predicts *foci* yield larger than the yield calculated by Belov et al. The main difference comes from estimation of irreparable fraction. In this work, the irreparable fraction is simulated as fraction of complex DNA damage ($\sim$ 0.12), on the other hand, the fraction used in the calculated yield was estimated directly as *foci* yield fraction at 24 h measured by Asaithamby et al. (0.01). Hence, it’s not surprising that the previous calculations are close to the experimental data at 24 h. It ought to be noted that, at such low yield, signal to noise ratio of the measurement is not high, since the intensity of signal light cannot be clearly separated from the background. Hence, experimentally, the systematic uncertainty of the measured *foci* yield at 24 h can be large. And due to background subtraction, detected *foci* yield can be lower than actual yield level, especially at such low yield. In the previous work by Belov et al.^15^, the model is able to predict yield of $\gamma$-H2AX foci in human skin fibroblasts (HSF42) irradiated with $^{16} \hbox {O}$, $^{28} \hbox {Si}$, and $^{56} \hbox {Fe}$. However, further investigations are required to make sure whether the application can predict yields of $\gamma$-H2AX foci for the other irradiation sources (including proton). In addition, the model parameters of biological repair, in this work, have been optimized for a human fibroblast cell. However, biological response against radiation and kinetics of DNA repair protein accumulation depend on cell type, since the repair path frequency, repair speed and its consequence varies from one cell line to another. For instance, application of the quantitative model to reconstruct the repair kinetics in cancer cells like breast carcinoma MCF-7 cells and NSCLC (lung) HTB177 cells^78^, requires a deeper understanding of the particular stages of DSB repair in such cells and, consequently, another set of parameters are needed. It is clear that in order to explore the mechanism of radiation induced DNA damage, the biological prediction model will need to be optimized cell line by cell line. We note, to simulate particle transport in a substrate, the properties of the substrate should be known, such as composition of material, density and mean excitation energy. Hence, for more accurate simulations, these specific properties should be provided to compare the simulations with the experimental studies. Nevertheless in this work, we place a water absorber substituting a substrate as an alternative approach.

## Conclusion

As a new important step for radiobiological simulation using Geant4-DNA, the first fully integrated simulation chain adopting IRT has been upgraded across the physical, chemical and biological stages of cellular radiation action in a single application. We have re-optimized parameters in the simulations including a more realistic cell geometry, whereby the cell nucleus is covered by a water absorber acting as cytoplasm.

To show the reliability of the application, we have simulated not only yield of DSBs for a mammalian cell but also the yield of SSB/DSB for plasmid simultaneously, as well as scavengeable DSB yields by switching off the histone scavenging functionality. Using this application, it now becomes possible to predict biological response along time through track-structure MC simulations. The simulation results for mammalian cells are in agreement with experimental data in terms of DSB yields (within 13.3% for protons on average and 0.6% for gamma rays from a $^{60} \hbox {Co}$ source) and $\gamma$-H2AX yields (within 1.6% on average for gamma rays from a $^{137} \hbox {Cs}$ source).

## Supplementary information

Supplementary Figure S1.
