# Supplementary material for: Fully integrated Monte Carlo simulation for evaluating radiation induced DNA damage and subsequent repair using Geant4-DNA
Source: Sci Rep. 2020 Nov 27;10:20788. doi: 10.1038/s41598-020-75982-x (PMC7695857; doi:10.1038/s41598-020-75982-x)
Supplement: Supplementary file 1 — Supplementary Figure S1. [file 41598_2020_75982_MOESM1_ESM.pdf]

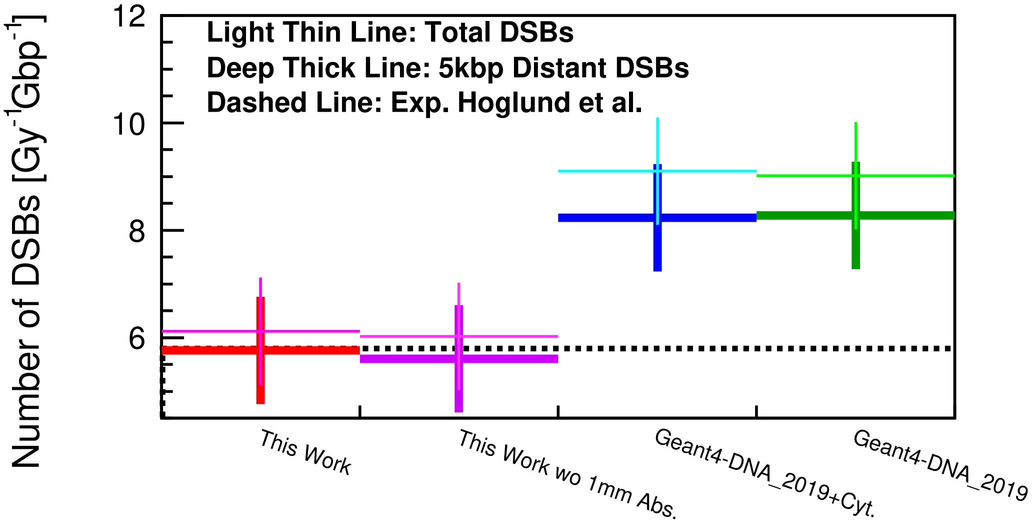

FIG. S.1. DSB yields and 5kbp distant DSB yields per Gy and per Gbp with different simulation conditions. The cell is irradiated with gamma rays emitted by a ^{60}Co source (energy equal to 1.17 MeV and 1.33 MeV with the same frequency). This Work: simulation condition of this work. This Work wo 1mm Abs.: simulation condition of this work without 1 mm thick absorber located downstream of beam. Geant4-DNA_2019+Cyt: simulation condition of Geant4-DNA_2019 with water absorber surrounding cell nucleus as cytoplasm geometry. Geant4-DNA_2019: simulation condition of Geant4-DNA_2019 without any geometry surrounding the naked cell nucleus. The diameter of incident particle plane is 28.0 μm for This Work. For the other conditions, the diameter is set as 14.2 μm.
